# Use of drugs for hypertension or heart failure and the risk of death in COVID-19: association with loop-diuretics

**DOI:** 10.1007/s00228-024-03709-2

**Published:** 2024-06-24

**Authors:** Johan Fastbom, Gudrun Jonasdottir Bergman, Johanna Holm, Håkan Hanberger, Kristoffer Strålin, Sten Walther, Joakim Alfredsson, Maria State, Natalia Borg, Anastasia Nyman Iliadou

**Affiliations:** 1 National Board of Health and Welfare (Socialstyrelsen), Stockholm, Sweden; 2 Aging Research Center, Karolinska Institutet and Stockholm University, Stockholm, Sweden; 3 Department of Clinical and Experimental Medicine, Infectious Diseases, Faculty of Health Sciences, Linköping University, Linköping, Sweden; 4 Department of Infectious Diseases, Karolinska University Hospital, Stockholm, Sweden; 5 Department of Cardiothoracic and Vascular Surgery. Heart Centre, Linköping University Hospital and Department of Health, Medicine and Caring Sciences, Linköping University, Linköping, Sweden; 6 Department of Health Medicine and Caring Sciences and Department of Cardiology, Linköping University, Linköping, Sweden

**Keywords:** COVID-19, Loop diuretics, Thiazide diuretics, Angiotensin-converting enzyme inhibitors

## Abstract

**Purpose:**

To study the association between the use of drugs for hypertension or heart failure, particularly diuretics, and risk of death in COVID-19.

**Methods:**

We conducted a cohort study, based on record linked individual-based data from national registers, of all Swedish inhabitants 50 years and older (*n* = 3,909,321) at the start of the first SARS-CoV-2 wave in Sweden. The association between use of angiotensin-converting enzyme inhibitors (ACEI), angiotensin II receptor blockers (ARB), thiazides, loop diuretics, aldosterone antagonists, beta blocking agents and calcium channel blockers at the index date 6 March 2020, and death in COVID-19 during 7 March to 31 July 2020, was analysed using Cox-proportional hazards regression, adjusted for a wide range of possible confounders.

**Results:**

Use of loop diuretics was associated with higher risk [adjusted hazard ratio (HR) 1.26; 95% confidence interval (95% CI) 1.17–1.35] and thiazides with reduced risk (0.78; 0.69–0.88) of death in COVID-19. In addition, lower risk was observed for ACEI and higher risk for beta-blocking agents, although both associations were weak. For ARB, aldosterone antagonists and calcium channel blockers no significant associations were found.

**Conclusion:**

In this nationwide cohort of nearly 4 million persons 50 years and older, the use of loop diuretics was associated with increased risk of death in COVID-19 during the first SARS-CoV-2 wave in Sweden. This contrasted to the decreased risk observed for thiazides. As treatment with loop diuretics is common, particularly in the elderly, the group most affected by severe COVID-19, this finding merit further investigation.

**Supplementary Information:**

The online version contains supplementary material available at 10.1007/s00228-024-03709-2.

## Introduction

The severe acute respiratory syndrome coronavirus 2 (SARS-CoV-2) attaches to its target cells through the membrane-bound angiotensin-converting enzyme 2 (ACE2). ACE2 has a key role in the renin–angiotensin–aldosterone system (RAAS) that regulates blood pressure and fluid and electrolyte balance.

Drugs that inhibit the RAAS-system, i.e. angiotensin-converting enzyme inhibitors (ACEI) and angiotensin II receptor blockers (ARB), increase the levels of ACE2 and it has therefore been suggested that treatment with these drugs might affect the risk of COVID-19 following exposure to SARS-CoV-2. Several studies have investigated whether treatment with ACEI or ARB is associated with risk for or severity of COVID-19, or hospitalisation, admission to an intensive care unit (ICU) or death, in COVID-19 patients. However, the results are conflicting, showing both an increased risk [1, 2], decreased risk [3–10], and no association with risk [11–14].

Some studies have also examined other medications used for the treatment of hypertension or heart failure; one reporting lower odds of COVID-19 diagnosis with calcium channel blockers but no significant association for beta blocking agents [8], one showing a better clinical outcome in COVID-19 with beta-blocking agents and a poorer outcome with calcium channel blockers [13], and one demonstrating a better clinical outcome for both beta blocking agents and calcium channel blockers [5]. The possible impact of different types of diuretics on the risk for, or outcome of, COVID-19 has, however, not been extensively investigated. Rezel-Potts et al. [8] reported lower odds for COVID-19 diagnosis and Hippisley-Cox et al. [15] a decreased risk for severe COVID-19 disease, with thiazides. In contrast, Mancia and colleagues [12] observed that the use of loop diuretics was associated with an increased risk for COVID-19.

During the first wave of the SARS-CoV-2 infection, we conducted a descriptive register-based study at the Swedish National Board of Health and Welfare (NBHW), examining the use before the start of the pandemic, of a broad range of drug groups, in persons who were later treated in ICU for, or dying from, COVID-19. In the study, we found a remarkable over-representation of the use of loop diuretics in these persons, compared to the Swedish population in the same age groups (18–69 and ≥ 70 years) [16]. Although this was a purely descriptive study that did not take comorbidity and other possible confounders into account, we considered that the result deserved further analysis.

Therefore, the aim of this nationwide register-based cohort study was to investigate the association between the use of drugs commonly prescribed for the treatment of hypertension or heart failure — ACEI, ARB, beta-blocking agents, calcium channel blockers and various types of diuretics including thiazides, loop-diuretics and aldosterone antagonists (mineralocorticoid receptor antagonists) — and the risk of death in COVID-19 in people 50 years or older, during the first SARS-CoV-2 infection wave in Sweden.

## Methods

We conducted a cohort study, based on record linked individual-based data from national registers hosted by the Swedish National Board of Health and Welfare (NBHW) and Statistics Sweden (SCB). The population consisted of all persons 50 years or older, with a valid personal identity number (PIN), registered as living in Sweden as of 31 December 2019, who were alive at the index date 6 March 2020 (the date when the first patient in Sweden was admitted to an ICU due to COVID-19). The age limit of 50 years was chosen as this group constitutes the vast majority of users of these drugs.

Information on age, sex and country of birth was obtained from the Total Population Register (hosted by SCB). Diagnoses were obtained from the Swedish National Patient Register, containing information on all reported cases of inpatient care and specialized outpatient care in Sweden (NBHW). Data on drug use were derived from the Swedish National Prescribed Drug Register (SPDR) containing detailed information on all prescribed and dispensed drugs (NBHW). Information on the date of death and underlying cause of death was obtained from the Swedish National Cause of Death Register (NBHW). Data about housing, home care and the number of home care hours were retrieved from the Swedish National Register for Care and Social Services for the Elderly and for Persons with Impairments (NBHW). Information on education and income was collected from the Longitudinal Integrated Database for Health Insurance and Labour Market Studies (LISA) register (SCB).

The studied outcome was death with COVID-19 as the reported underlying cause, during the period 7 March to 31 July 2020. The following exposures were studied: the use of drugs within each of the drug groups [ATC-codes (Anatomical Therapeutic Chemical classification system; WHO) within parentheses] ACEI (C09A, C09B), ARB (C09C, C09D), thiazides (C03A, C03EA, C09BA, C09DA), loop diuretics (C03C), aldosterone antagonists (C03DA), beta blocking agents (C07) and calcium channel blockers (C08, C07FB, C09BB, C09DB). The current use of these drugs at the index date was estimated based on information about the date of drug dispensing at the pharmacy, the amount of drug dispensed and the prescribed dosage, for drugs dispensed within a 3-month interval before the measurement date, according to a previously described method [17]. Cox-proportional hazards regression models were used to analyse the association between drug use and death in COVID-19. Adjustments were made for a range of possible confounders: age group (50–59, 60–69, 70–79, 80–89 and ≥ 90 years), sex, country of birth, home county, big city (city with at least 200,000 inhabitants) dwelling, education, income, comorbidity, living in retirement home, presence of home care, number of home care hours and number of other drugs. Measures of co-morbidity consisted of the diagnoses regarded as potential risk factors for severe COVID-19 disease: cardiovascular disease, hypertension, asthma, lung disease, diabetes, kidney disease/failure, liver disease, malignancy, dementia, obesity, neurological disease and immunodeficiency (Supplementary Table S1). These diagnoses were measured 5 years back in time from the index date. In addition, the analysis of each drug group was adjusted for the use of the other studied drug groups. Moreover, to further refine each analysis, we excluded persons with seemingly concurrent use of drug groups with similar therapeutic effects. This exclusion was done to avoid dilution of the effect of the drug under study by adding the effect of the concurrent drug to the denominator. Thus, for the analysis of ACEI and ARB, persons with concurrent use of these two types of drugs were excluded and in the analysis of the three types of diuretics, persons with concurrent use of any of the other two types of diuretics were excluded.

## Results

Table 1 describes the study population. The mean age was considerably higher among patients who died in COVID-19 (83 years) compared to the total study population (66 years), mainly due to a higher proportion of persons 80 years and older. Those who died in COVID-19 were also more likely to be male, have a lower education, be born outside of Sweden, live in a retirement home and to have polypharmacy (use of five or more drugs). They also had a higher prevalence of the majority of comorbid conditions studied.

**Table 1:** Description of the study population

**Variables**	**All** ***n*** = 3 909 321	**Dead in COVID-19** ***n*** = 5 799	***P*-value**^**a**^
Age, mean (SD)	66.3 (11.0)	82.6 (9.7)	< 0.0001
Age groups, *n* (%)			< 0.0001
50–59	1 295 671 (33.1)	170 (2.9)	
60–69	1 104 669 (28.3)	416 (7.2)	
70–79	983 785 (25.2)	1 290 (22.2)	
80–89	430 052 (11.0)	2 488 (42.9)	
≥ 90	95 144 (2.4)	1 435 (24.7)	
Male sex, *n* (%)	1 892 287 (48.4)	3 129 (54.0)	< 0.0001
Education, *n* (%)			< 0.0001
Compulsory school	865 377 (22.1)	2 393 (41.3)	
Secondary school	1 746 955 (44.7)	2 139 (36.9)	
University	1 251 829 (32.0)	1 045 (18.0)	
Country of birth, *n* (%)			< 0.0001
Sweden	3 254 064 (83.2)	4 532 (78.2)	
Europe excl. Sweden	403 540 (10.3)	796 (13.7)	
Other	251 717 (6.4)	471 (8.1)	
Big city dwelling, *n* (%)	1 201 628 (30.7)	2 767 (47.7)	< 0.0001
Retirement home resident, *n* (%)	86 799 (2.2)	2 616 (45.1)	< 0.0001
Comorbidites, *n* (%)			
Cardiovascular disease	552 332 (14.1)	2 875 (49.6)	< 0.0001
Hypertension	1 106 545 (28.3)	3 521 (60.7)	< 0.0001
Heart failure	141 213 (3.6)	1 268 (21.9)	< 0.0001
Asthma	87 983 (2.3)	263 (4.5)	< 0.0001
Lung disease	102 669 (2.6)	640 (11.0)	< 0.0001
Diabetes	284 749 (7.3)	1 395 (24.1)	< 0.0001
Kidney disease/failure	68 026 (1.7)	772 (13.3)	< 0.0001
Liver disease	24 974 (0.6)	80 (1.4)	< 0.0001
Malignancy	268 856 (6.9)	798 (13.8)	< 0.0001
Dementia	59 975 (1.5)	1 472 (25.4)	< 0.0001
Overweight and obesity	64 932 (1.7)	171 (2.9)	< 0.0001
Neurological disease	126 813 (3.2)	1 232 (21.2)	< 0.0001
Immunodeficiency	2 155 (0.1)	8 (0.1)	< 0.05
Number of drugs, mean (SD)	4.5 (3.5)	8.3 (4.4)	< 0.0001
Use of ≥ 5 drugs, *n* (%)	1 024 248 (26,2)	4 342 (74,9)	< 0.0001
Drug use, *n* (%)			
ACE-inhibitors	442 129 (11.3)	1 054 (18.2)	< 0.0001
Angiotensin II-antagonists	616 346 (15.8)	1 097 (18.9)	< 0.0001
Thiazides	362 903 (9.3)	365 (6.3)	< 0.0001
Loop-diuretics	169 703 (4.3)	1 713 (29.5)	< 0.0001
Aldosterone antagonists	78 412 (2.0)	330 (5.7)	< 0.0001
Beta blocking agents	695 692 (17.8)	2 362 (40.7)	< 0.0001
Calcium channel blockers	561 536 (14.4)	1 194 (20.6)	< 0.0001

^a^By chi-square (categorical variables) and Mann–Whitney *U* tests (continuous variables)

**Fig. 1:**
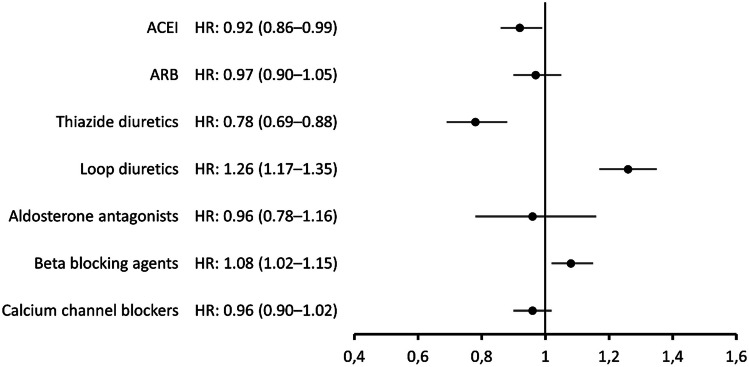
Forest plot showing the association between the use of medicines for hypertension or heart failure at index date and the risk of dying in COVID-19. Adjusted hazard ratio (HR) with 95% confidence intervals. ACEI, angiotensin-converting enzyme inhibitors; ARB, angiotensin II receptor blockers

The adjusted Cox-proportional hazards regression analysis showed that the use of thiazides, and to some extent ACEI, was associated with a decreased risk of dying in COVID-19. In contrast, persons who used loop diuretics showed a nearly 30% higher risk of dying in COVID-19 (Fig. 1, Supplementary Table S2). A slightly increased risk was also observed for beta-blocking agents. For ARB, aldosterone antagonists and calcium channel blockers no significant associations were found.

As we in these analyses excluded persons with seemingly concurrent use of drug groups with similar therapeutic effects, we examined the impact of this measure on our results, by repeating the analyses without any exclusion. This gave HR point estimates that were slightly closer to 1.00 for loop diuretics: 1.25 (95% CI 1.17–1.34) thiazides: 0.80 (0.71–0.88) and ACEI: 0.95 (0.89–1.02). For ACEI this meant that the result was not significant.

As one common indication for the use of loop-diuretics is oedema in heart failure and this diagnosis (ICD-10 code I50) was severalfold (six times) more common in persons dying with COVID-19, we further examined the association with loop-diuretics by stratifying the analysis by heart failure and found that the HR for loop-diuretics was 1.30 (95% CI 1.20–1.41) in persons without and 1.15 (1.00–1.32) in persons with heart failure diagnosis. For the same reason, we stratified the analysis by the diagnosis of kidney disease/failure and found that the HR for loop-diuretics was 1.32 (1.22–1.42) in persons without and 1.12 (0.96–1.32) in persons with this diagnosis.

When the same stratification was made for thiazides, the HR was 0.79 (95% CI 0.70–0.90) in persons without and 0.80 (0.48–1.25) in persons with heart failure diagnosis. For kidney disease/failure, the HR was 0.80 (0.70–0.91) in persons without and 0.95 (0.61–1.42) in persons with the diagnosis.

## Discussion

In this study, we have used nationwide record linked register data from nearly four million persons 50 years and older, to assess the association between the use of different types of drugs used to treat hypertension or heart failure, and the risk of death in COVID-19 during the start of the first SARS-CoV-2 wave in Sweden. The main findings were that the use of loop diuretics was associated with a higher risk of death in COVID-19, while the use of thiazide diuretics was associated with a reduced risk. We also observed a slightly lower risk of death with ACEI and a slightly higher risk with beta-blocking agents.

Our results support previous findings that people who use ACEI or ARB are not at higher risk of dying in COVID-19 [4–6, 9–11, 13]. Instead, our results for ACEI suggest, although the association was weak, that these drugs may have a protective effect against poor outcome in COVID-19, also in agreement with a number of studies [4–6, 9, 10].

However, a more pronounced negative association with the risk of death in COVID-19 was observed for thiazide diuretics. This is in line with two previous studies reporting associations between thiazide use and a lower risk for COVID-19 [8] and severe COVID-19 disease [15].

The other main finding of the present study was that people who used loop diuretics were at higher risk of death in COVID-19. In one earlier descriptive study at the NBHW, we found loop diuretics to be one of the drug groups being most over-represented among persons who were either treated in ICU for, or died from, COVID-19, compared to the general population in the same age range [16]. This led us to hypothesise that the use of loop diuretics may increase the risk of COVID-19 with poor outcome. This hypothesis is supported by the present study, where the association with loop diuretics was analysed taking a wide range of possible confounders into account.

Guragai and colleagues investigated the effects of baseline diuretic use on the prognosis in patients admitted to hospital with COVID-19 and found no significant difference between users and non-users, in COVID-19 severity or mortality [18]. However, their study was small and did not differentiate between the different types of diuretics. To our knowledge, only one study has specifically examined the association between the use of loop diuretics and the outcome in COVID-19. This was a population-based case–control study from the Italian Lombardy region, on 6272 patients with confirmed COVID-19 and 30,759 matched controls [12]. They found no significant association with risk for COVID-19 overall, or among patients who had a severe or fatal course of the disease, for ACEI, ARB, beta-blocking agents, calcium channel blockers, thiazides or aldosterone antagonists. By contrast, an increased risk was observed for loop-diuretics (OR 1.46; 95% CI 1.23–1.73). The authors judged, however, that this finding was likely to be explained by the fact that the use of loop diuretics reflects the existence of clinical conditions such as heart failure or advanced renal damage, rather than an effect of the drugs per se.

The present study largely corroborates their findings [12], pointing towards an increased risk of death in COVID-19 in users of loop-diuretics, and this was observed in an analysis adjusted for a broad range of possible confounders, including heart failure and kidney disease/failure.

Moreover, as regards heart failure, we obtained an even stronger result in patients without this diagnosis. One may also argue that, if the presence of heart failure would explain the association with loop diuretics, some of the other drugs that are more closely related to treatment of heart failure, i.e. ACEI, ARB, beta blocking agents and aldosterone antagonists, would have been associated with an increased risk in our analysis. We did observe a positive association with beta-blocking agents, indicating an increased risk, but this was weak and for the other drug groups no such association was seen.

As to advanced renal failure, this diagnosis is reportedly uncommon among users of loop diuretics, compared to heart failure and other cardiovascular diseases [19]. Further, similar to the results for heart failure, we observed a higher HR for loop diuretics in patients without a diagnosis of kidney disease/failure.

Clearly, as the present study is observational, it cannot prove causality and we cannot exclude the possibility of confounding by factors that have not been accounted, or properly adjusted, for in the analysis. However, if our results would reflect a causal relationship between the use of loop diuretics and increased risk of death in COVID-19, there are several plausible mechanisms for this. Saheb Sharif-Askari and colleagues [20] examined the effects of common drugs, including the loop diuretic furosemide, on the expression of SARS-CoV-2 entry receptors in kidney tissue. They did not find that furosemide increased the expression of ACE2, but instead the opposite. However, although this could reduce the entry of the SARS-CoV-2 virus, a downregulation of ACE2 may still worsen the prognosis in COVID-19, as there is evidence that the ACE2 enzyme also has organ protective functions in hypertension, diabetes and cardiovascular disease, as well as against acute respiratory distress syndrome (ARDS) [21]. In fact, the SARS-CoV infection per se can downregulate ACE2 expression, which may contribute to multiple organ injury in COVID-19 [22, 23].

Moreover, the study by Saheb Sharif-Askari et al. [20] showed that furosemide was one of the drugs that increased the expression of the transmembrane protease serine 2 (TMPRSS2), as well as being the top upregulating drug for the transmembrane glycoprotein CD147. TMPRSS2 acts by priming the virus spike protein, necessary for the virus-host cell membrane fusion and cell entry [24] and CD147 has been shown to play a functional role in facilitating invasion of host cells by SARS-CoV [25, 26].

Another possible mechanism for an increased risk of death in COVID-19 in loop diuretic users is QTc-prolongation and the subsequent risk of ventricular arrhythmias. Studies have demonstrated that QTc-prolongation is more frequent in COVID-19 patients and may be important for the outcome of the disease. A single-centre cohort study of patients hospitalised with COVID-19 [27] found that 9.8% had a prolonged QTc-interval on admission, which was independently associated with a higher mortality, even after adjustment for treatment with hydroxychloroquine and azithromycin. Another single-centre cohort study, of patients admitted to the hospital for COVID-19, showed that the QTc-intervals on presentation with COVID-19 were longer than in ECGs taken before the disease. This was seen in both those who survived and died with COVID-19, but the prolongation was more marked in the deceased patients [28].

The use of loop diuretics may be of importance for this QTc-prolongation. In a systematic review of 10 observational studies, Vandael et al. [29] found that the use of diuretics was — together with hypokalaemia, antiarrhythmic drugs and specific QTc-prolonging drugs — a factor with ‘very strong evidence’ for being a risk factor for QTc-prolongation. Moreover, in an earlier study of 900 consecutive patients admitted to cardiac care units, loop diuretics were found to be one of the independent predictors of QTc prolongation [30].

It is interesting to note that the two types of diuretic drugs — thiazides and loop diuretics — were found to have opposite associations with death in COVID-19 in our analysis. Our present results, suggesting a potential protective effect of thiazides, are in line with previous findings. Hippisley-Cox et al. [15] observed a decreased risk with thiazides for severe COVID-19 disease, while Rezel-Potts and colleagues [8] reported lower odds of COVID-19 diagnosis, but not mortality, in COVID-19, when using thiazides.

Our findings are further supported by a recent Spanish retrospective cohort study of 15,968 patients hospitalized with COVID-19. In the study, which aimed to find effective treatments among drugs used for other indications, 21 drugs out of a total of 864 treatments were found to be associated with improved patient survival, among them hydrochlorothiazide. In contrast, only one drug — the loop-diuretic furosemide — was associated with an increase in patient mortality [31].

The present results, suggesting that the use of loop diuretics may contribute to the risk of poor outcome in COVID-19, should be considered in light of the fact that these drugs are common in the elderly, who are also the age group most affected by severe COVID-19; and that a considerable proportion of these drugs are prescribed without a proper indication, in elderly patients [19].

The present study covers the first SARS-CoV-2 infection wave in Sweden, when no vaccine was available and the knowledge about how to treat patients with COVID-19 was limited. Since then, better treatments — such as corticosteroid, anticoagulant and more recently antiviral therapy — are provided and since December 2020 a high proportion of Swedish citizens have been vaccinated. Moreover, numerous new mutations of the virus have emerged. All this may have changed the possible impact of medications on the risk of serious outcomes of COVID-19, something that should be explored in further studies.

### Strengths and limitations

The strength of this study lies in the large, nationwide cohort-based data from multiple, record linked national registers. This favours the generalisability of the results. However, there are also some limitations. One is the lack of information on persons with COVID-19 who did not die from the disease. For several reasons, only few of them could have been diagnosed during the study period. First, most likely a considerable number of COVID-19 cases were asymptomatic or showed symptoms or signs that did not lead to suspicion of COVID-19. Second, at this early stage of the pandemic, testing for COVID-19 in the Swedish population was very limited and restricted in essence to severe cases.

Other limitations are related to the register-based design of the study. First, as the study is based on register data, we lack more detailed clinical data on the studied persons, such as physiological and laboratory parameters. Second, the registers used have some shortcomings. The SPDR only records prescription drugs dispensed at pharmacies. Over-the-counter drugs and drugs administered in hospitals or from drug storerooms in nursing homes are not recorded in the register, potentially leading to an underestimation of the actual drug exposure. However, between 80 and 90% of DDDs of drugs dispensed to the Swedish population annually are prescription drugs delivered through pharmacies and are therefore covered by the SPDR. The patient register, providing the data on diagnoses, only covers inpatient and specialised outpatient care but not primary care. Therefore, some of the diagnoses processed as confounders in our analyses, particularly those often treated in primary care, such as diabetes, hypertension, dementia and obesity, may not have been fully covered.

## Conclusion

Using nationwide record linked register data from persons 50 years and older in Sweden, we found an association between the use of loop diuretics at the start of the first SARS-CoV-2 wave in Sweden and risk of death in COVID-19 during this wave. In contrast, a negative association, indicating a decreased risk, was observed for thiazide diuretics and to some extent ACE inhibitors. The association with loop diuretic use might have several conceivable mechanisms. As loop diuretics are common, particularly in the elderly, who are also the group most affected by severe COVID-19, this finding merits further investigation.

## Supplementary Information

Below is the link to the electronic supplementary material.

Supplementary file1 (DOCX 28 KB)

## Data Availability

The data used for the analyses are based on individual data which cannot be shared due to confidentiality, in accordance with Swedish law and the European Union General Data Protection Regulation (GDPR). However, upon reasonable request, additional analyses can be conducted after contact with the corresponding author.
