# Supplementary material for: Use of drugs for hypertension or heart failure and the risk of death in COVID-19: association with loop-diuretics
Source: Eur J Clin Pharmacol. 2024 Jun 24;80(10):1515–22. doi: 10.1007/s00228-024-03709-2 (PMC11393138; doi:10.1007/s00228-024-03709-2)
Supplement: Supplementary file 1 — Supplementary file1 (DOCX 28 KB) [file 228_2024_3709_MOESM1_ESM.docx]

Use of drugs for hypertension or heart failure and the risk of death in COVID-19: association with loop-diuretics

Johan Fastbom, Gudrun Jonasdottir Bergman, Johanna Holm, Håkan Hanberger, Kristoffer Strålin, Sten Walther, Joakim Alfredsson, Maria State, Natalia Borg, Anastasia Nyman Iliadou

Corresponding author: Johan Fastbom, National Board of Health and Welfare, Sweden [johan.fastbom@socialstyrelsen.se](mailto:johan.fastbom@socialstyrelsen.se)

**Table S1.** Definition of covariates according to ICD-10^1^ and ATC^2^ codes.

| Diagnosis | Definition |
| --- | --- |
| Cardiovascular disease | I20-I25, I48, I50, I61, I63, I649, I65, I691, I693, I694, I698, I70 |
| Hypertension | I10-I15, combination of two or more drugs with the following ATC-codes: I. Antiadrenergic agents (C02A, C02B, C02C), II. Non-loop diuretics (C02DA, C02L, C03A, C03B, C03D, C03E, C03X, C07C, C07D, C08G, C09BA, C09DA, C09XA52), III. Vasodilators (C02DB, C02DD, C02DG, C04), IV. Beta blocking agents (C07), V. Calcium channel blockers (C07FB, C08, C09BB, C09DB), VI. Agents acting on the renin-angiotensin system (C09). |
| Asthma | J45, J46 |
| Lung disease | J40-J44, J47, J60-J67, J684, J701, J703, J961, E840 |
| Diabetes | E10-E14, ATC: Drugs used in diabetes (A10) |
| Kidney disease/failure | N18, N19 |
| Liver disease | K70, K71, K721, K729, K73-K77 |
| Malignancy | C00-C97, Z85 |
| Dementia | F00-F02, F039, F107A, G30 |
| Obesity | E66 |
| Neurological disease | G10-G14, G20-G26, G30-G32, G35-G37, G70-G73, G80-G83 |
| Immunodeficiency | D80 |

^1^ International Classification of Diseases 10th version

^2^ Anatomical Therapeutic Chemical classification system

**Table S2.** Use of medicines for hypertension or heart failure at index date and the risk of dying in COVID-19. The risk is expressed as hazard ratio, HR, with 95% confidence intervals, CI.

| Drug group | All, n | Dead in  COVID-19, n | Crude  HR (95% CI) | Adjusted^1^  HR (95% CI) |
| --- | --- | --- | --- | --- |
| ACEI | 435 226 | 1 043 | 1.88 (1.75–2.01) *** | 0.92 (0.86–0.99) * |
| No ACEI | 2 857 749 | 3 659 |  |  |
| ARB | 609 443 | 1 086 | 1.39 (1.30–1.49) *** | 0.97 (0.90–1.05) |
| No ARB | 2 857 749 | 3 659 |  |  |
| Thiazide diuretics | 341 215 | 300 | 0.80 (0.71–0.90) *** | 0.78 (0.69–0.88) *** |
| No thiazide diuretics | 3 347 166 | 3 676 |  |  |
| Loop diuretics | 130 618 | 1 441 | 10.33 (9.71–10.97) *** | 1.26 (1.17–1.35) *** |
| No loop diuretics | 3 347 166 | 3 676 |  |  |
| Aldosterone antagonists | 42 577 | 102 | 2.20 (1.79–2.66) *** | 0.96 (0.78–1.16) |
| No Aldosterone antagonists | 3 347 166 | 3 676 |  |  |
| Beta blocking agents | 695 692 | 2 362 | 3.19 (3.03–3.37) *** | 1.08 (1.02–1.15) * |
| No beta blocking agents | 3 213 629 | 3 437 |  |  |
| Calcium channel blockers | 561 536 | 1 194 | 1.55 (1.45–1.65) *** | 0.96 (0.90–1.02) |
| No Calcium channel blockers | 3 347 785 | 4 605 |  |  |

^1^Adjusted for age, sex, country of birth, home county, big city dwelling, education, income, comorbidity, living in retirement home, presence of home care, number of home care hours, other studied drug groups (see method description), number of other drugs.

****p*<0.001, ***p*<0.01, **p*<0.05
